# The specialty choices of graduates from Brighton and Sussex Medical School: a longitudinal cohort study

**DOI:** 10.1186/s12909-015-0328-z

**Published:** 2015-03-13

**Authors:** Katherine Woolf, Caroline Elton, Melanie Newport

**Affiliations:** 1 University College London Medical School (UCLMS), Room GF/664, Royal Free Hospital, London, NW3 2PF UK; 2 London Deanery, The Careers Unit, Room ST201, Stewart House, 32 Russell Square, London, WC1B 5DN UK; 3 Brighton & Sussex Medical School, BSMS Teaching Building, University of Sussex, Brighton, East Sussex BN1 9PX UK

**Keywords:** Careers, Specialty choice, Longitudinal, Experience

## Abstract

**Background:**

Since 2007 junior doctors in the UK have had to make major career decisions at a point when previously many had not yet chosen a specialty. This study examined when doctors in this new system make specialty choices, which factors influence choices, and whether doctors who choose a specialty they were interested in at medical school are more confident in their choice than those doctors whose interests change post-graduation.

**Methods:**

Two cohorts of students in their penultimate year at one medical school (n = 227/239) were asked which specialty interested them as a career. Two years later, 210/227 were sent a questionnaire measuring actual specialty chosen, confidence, influence of perceptions of the specialty and experiences on choice, satisfaction with medicine, personality, self-efficacy, and demographics. Medical school and post-graduation choices in the same category were deemed ‘stable’. Predictors of stability, and of not having chosen a specialty, were calculated using bootstrapped logistic regression. Differences between specialties on questionnaire factors were analysed.

**Results:**

50% responded (n = 105/277; 44% of the 239 Year 4 students). 65% specialty choices were ‘stable’. Factors univariately associated with stability were specialty chosen, having enjoyed the specialty at medical school or since starting work, having first considered the specialty earlier. A regression found doctors who chose psychiatry were more likely to have changed choice than those who chose general practice. Confidence in the choice was not associated with stability. Those who chose general practice valued lifestyle factors. A psychiatry choice was associated with needing a job and using one’s intellect to help others. The decision to choose surgical training tended to be made early. Not having applied for specialty training was associated with being lower on agreeableness and conscientiousness.

**Conclusion:**

Medical school experiences are important in specialty choice but experiences post-graduation remain significant, particularly in some specialties (psychiatry in our sample). Career guidance is important at medical school and should be continued post-graduation, with senior clinicians supported in advising juniors. Careers advice in the first year post-graduation may be particularly important, especially for specialties which have difficulty recruiting or are poorly represented at medical school.

## Background

In the United Kingdom (UK), medical school is typically a five year undergraduate course following from secondary school. Upon graduation from medical school, junior doctors have the opportunity to work in a variety of specialties before choosing one for further training. In 2005, the Modernising Medical Careers (MMC) reforms to postgraduate medical training were introduced. Junior doctors now have two years Foundation Training immediately after medical school and have to choose a specialty in their second Foundation year (FY2) i.e. 18 months after finishing medical school. Given that prior to MMC about a quarter of doctors had not decided on their final specialty at that point [1], there is concern that 18 months is too soon to make longer term specialty decisions.

The Independent Inquiry into Modernising Medical Careers conducted by Sir John Tooke (‘The Tooke Report’) [2] was set up to investigate some of MMC’s initial difficulties. It found that many junior doctors felt that the FY2 year was too early to decide on a specialty, particularly as they may not have been exposed to many by that point. With exposure opportunities reduced, medical school experiences become more important. The Tooke Report recommends medical schools play a greater role in preparing students for career decisions. This seems prudent as a recent survey of 115 final year medical students at a London medical school found 15% chose ‘don’t know’ as their career option out of a list of 15 broad groups and this option was the second most popular after general practice (family medicine) [3]. A study at Liverpool medical school found that about 20% of final year medical students did not know what specialty to choose [4].

Specialty choices are often influenced by medical school experiences. Goldacre and colleagues [1] asked a large number of junior doctors one to two years after graduation in 2000 and 2001 to state up to three long term career choices, and asked which factors influenced their choice ‘a great deal’. Nearly half said their experience as a student, about a quarter said a particular teacher or department, and about a sixth said experience prior to medical school. Experiences can inform career choice because they are positive or negative. A small pre-MMC longitudinal study of graduates of a UK medical school found that about half of junior doctors in their first or second year post-graduation expressed a strong interest in the same specialty they had previously expressed a strong interest in during their fourth year of medical school, and less than one percent was strongly attracted to a specialty they had been very uninterested in at medical school [5].

Specialty choice can also be influenced by a other factors, including gender [6], personality [7], perceptions about job-related factors such as work-life balance [8], not to mention unexpected opportunity and luck; and as such, trying to predict medical careers is notoriously difficult [9]. The more that is known about the factors that underpin specialty choice however, the more it may be possible to point out these key factors to medical students and junior doctors as part of careers support activities that help this group make robust decisions.

The current study followed two cohorts of Brighton and Sussex Medical School (BSMS) graduates, who had completed a formative career planning exercise during Year 4 of medical school. They were followed up at the point that they made their specialty applications in their second Foundation Year (FY2). We aimed to compare graduates’ Year 4 specialty interests with their self-reported specialty application choices (or lack thereof) and to investigate which psychological, educational, and demographic factors related to stability of specialty choice. Research questions were:

1. Do graduates’ specialty choices change from Year 4 to Foundation Year 2 (FY2)?
2. Which experiential, psychological and demographic factors relate to choice of specialty and stability of choice over time?
3. Is stability of choice related to confidence in choice?
4. Which factors predict not having chosen a specialty at FY2?

## Methods

### Participants and procedure

In 2007 and 2008, Year 4 students at Brighton and Sussex Medical School (BSMS) completed a formative career planning exercise in which they wrote the names of two specialities they were interested in as a future career (see [10] for details).

In December 2009 and 2010 these students, now graduates, were emailed an invitation to complete an online questionnaire, the first page of which was a consent form (further details about the questionnaire below). To reduce bias [11] questionnaires were administered after the closing date for specialty applications had passed, but before shortlisting. Three email reminders were sent and non-respondents were telephoned once to check they had received the email. In 2010, 13 respondents were chosen at random to receive a £30 book voucher incentive.

### Questionnaire

The questionnaire measured which specialties participants had applied for, their preferred choice, their confidence that they would be successful in their preferred choice, and their confidence that their preferred choice was right for them.

To assess general satisfaction, participants were asked how often they had considered leaving medicine in the last year (never, once or twice, monthly, weekly or daily).

To explore the role of previous experience, participants were asked:

- their favourite and least favourite medical school placements
- how much 10 experiences had influenced their choice (3 point scale)
- whether they had been dissuaded from entering a specialty (5 point scale)
- when they had first considered and definitely decided on their specialty (before medical school, during medical school, during FY1, during FY2, still undecided)

Participants rated on a 3 point scale how much 23 aspects of their chosen specialty (including lifestyle factors, prestige, job security and extent of patient contact) had influenced their choice.

A 15-item version of a big 5 personality questionnaire [12,13] and a 10-item measure of general self-efficacy [14] were included.

The questionnaire was piloted on a convenience sample of junior doctors, and altered in light of their feedback.

### Coding of freetext specialties

Specialties were coded verbatim. 43 specialties were initially categorised into: hospital medical, paediatrics, accident and emergency, surgery, obstetrics and gynaecology, anaesthetics, radiology, clinical oncology, pathology, psychiatry, general practice, public health, and ‘other medical’[15]. Clinical oncology and ‘other medical’ were subsumed into hospital medical, and public health was expanded to included research. Anaesthetics and emergency medicine specialty training are covered by the specialty training path Acute Care Common Stem (ACCS), so ACCS, anaesthetics, and emergency medicine were combined into a single category. The final ten categories were: acute care, hospital medicine, surgery, obstetrics and gynaecology, psychiatry, general practice, paediatrics, pathology, public health and research, radiology. This broadly corresponds to the 15 specialties that graduates could apply to in 2011 [16].

### Statistical analysis

All analyses were performed in SPSS v20.

### Perceptions of specialty chosen

The 23 items measuring perceptions of the specialty choice were factor-analysed using a Varimax rotation. The scree plot indicated the presence of three to five factors. Three, four and five factor solutions were attempted, and a four factor solution chosen (See Table 1).

**Table 1:** **Rotated Component Matrix showing the loadings of each item on to the four aspects of a specialty that influenced choice**

Item name	Lifestyle	Money and status	Needed a job	Using intellect to help others
Suits my personality	0.525			0.439
Suits my skills & aptitude	0.287			0.495
Intellectually stimulating	−0.321	0.320		**0.667**
Change people’s lives				**0.613**
Opportunity for research				**0.659**
Right patient contact			0.204	**0.644**
New technologies	−0.274	0.558		0.321
Training opportunities		0.319	0.290	0.503
Easy to get job	0.419		**0.684**	
Needed job this year			**0.801**	
Good promotion		0.409	0.521	
Job security	0.477	0.574	0.281	
Can change specialty later		0.457	0.294	
Well paid	0.504	**0.692**		
Well regarded		**0.780**		0.262
Private practice		**0.701**		
Geographic location	0.539	0.379	0.276	
On call & shifts	**0.822**			
Family friendly	**0.878**			
Outside interests	**0.862**			
Job control	**0.853**			
Less discrimination		0.366	0.213	0.305
Best of bad bunch			**0.880**	

Higher numbers indicate a closer association between the item score and the factor score e.g. between “private practice” and “Money & Status” (items with loadings >0.6 are in bold). Negative signs indicate a high score on the item is associated with a low score on the factor.

**Factor 1** ‘Lifestyle’. High scoring individuals valued having a life outside of medicine, meeting family commitments, and job flexibility and autonomy.

**Factor 2** ‘Money and status’. High scoring individuals valued financial security and high social status.

**Factor 3** ‘Needed a job’. This was a factor relating to a negative choice. High scoring individuals were likely to have chosen a specialty they were likely to get a job in.

**Factor 4** ‘Using intellect to help others’. High scoring individuals valued intellectually stimulating careers in which they could make a difference to people’s lives, either directly via patient contact, or indirectly via research.

### Non-parametric testing and bootstrapping

Bootstrapping is a non-parametric statistical technique used to estimate the sampling distribution of a statistic by resampling with replacement many times the data from one sample [17]. Bootstrapping does not make assumptions about the population and can therefore be used when the sample is small and when assumptions of Normality are violated. Bootstrapping was used to estimate 95% confidence intervals (CI) for means and standard errors, and regression coefficients, as well as for medians and percentages, as described in the SPSS bootstrapping handbook [18]. Bootstrapping was typically performed using 1,000 random bootstrap samples. As a sensitivity test, and for ease of interpretation, conventional non-parametric statistical tests (Chi-squared, Mann Whitney U, and Kruskal Wallis) were also used. There results of both are presented.

### Stability of specialty choice from Year 4 to FY2

The stability of specialty choice from Year 4 of medical school to FY2 was the main outcome variable of interest. A categorical variable *Stability* was created [2 = at least one Year 4 specialty in the same category as the FY2 specialty (‘stable choice’); 1 = no Year 4 choices in the same category as the FY2 choice (‘different choice’); 0 = no specialty chosen at FY2].

Respondents with a ‘stable choice’ were compared to those with a ‘different choice’ (those who had not chosen a specialty were excluded).

### Differences between specialties

Respondents who had chosen different specialties at FY2 were compared (those who had not chosen a specialty were excluded).

### No specialty chosen at FY2

A *No Specialty* variable was created by combining ‘stable choice’ and ‘different choice’, and comparing this category to those who had not chosen a specialty.

Univariate analyses were conducted as follows:

- Categorical variables (cohort, specialty chosen, sex, ethnicity, when first considered specialty, when decided to apply for specialty): chi-squared tests were used to compare proportions in each group, and the bootstrapped 95% confidence intervals of proportions of respondents in each group were compared.
- Continuous variables (confidence, personality, general self-efficacy, perceptions of the specialty): bootstrapped independent t-tests and one-way ANOVAs, Mann Whitney U and Kruskal Wallis tests were used to compare groups.
- Ordinal variables with three levels (satisfaction with medicine as a career, previous experiences, whether dissuaded from entering a specialty): bootstrapped group medians were compared, and Mann Whitney U tests were also performed.
- Bootstrapped Pearson’s correlations were calculated to estimate the strength and direction of the relationships between personality, self-efficacy, and confidence.

Logistic regression was used to identify the independent predictors of the *Stability* and *No Specialty* variables from those factors found to be statistically significant in the univariate analyses. Bootstrapping was used to estimate p values and the 95% confidence intervals for the regression coefficients and standard errors.

### Ethical approval

Ethical approval for the study was granted by the UCL Ethics Committee (ref: 0511/004 and 0511/005).

## Results

### Sample

All 96 students in the 2007 Year 4 cohort completed the career planning exercise, and 43/96 (45%) responded to the questionnaire.

131/143 students in the 2008 Year 4 cohort completed the career planning exercise. Email addresses were available for 114/143, of who 62/114 (54%) responded to the questionnaire.

There were no significant differences between cohorts in stability of choice (*χ*2 = 0.03; df = 1; p = 0.857) and so the 2007 and 2008 cohorts were combined for analysis.

Of 239 Year 4 students, 227 completed the career planning exercise in Year 4, and 210 had email addresses for follow-up in FY2. Of those 210, 105 gave usable responses to the questionnaire, giving a response rate of 50% (44% of the 239 eligible to take part in the career planning exercise).

The sample of 105 comprised 36 men and 66 women (3 missing); 81/101 of white ethnicity, 11 of Asian ethnicity (including Chinese), 4 of mixed ethnicity, and 4 of black ethnicity (5 missing). The Asian, black, mixed, and Chinese groups were combined into a Black and Minority Ethnic (BME; n = 20) group for analysis. The age range was 24 to 41 years, median 25 (4 missing).

### Descriptive statistics

#### Year 4 specialty interests

The two Year 4 specialty interests chosen by each student were summed to give an overall number of times each specialty was selected (see Table 2).

**Table 2:** **The specialties selected, in no particular order, by respondents when they were in Year 4 of medical school (sum in parentheses)**

Specialty	N (sum of two year 4 interests)
Acute care common stem	14 + 10 (24)
General practice	23 + 27 (50)
Medical specialties	23 + 22 (45)
O&G	10 + 9 (19)
Paediatrics	11 + 6 (17)
Pathology	0
Psychiatry	5 + 7 (12)
Public health and research	2 + 2 (4)
Radiology	1 + 1 (2)
Surgical specialties	7 + 9 (16)
Missing	9 + 12 (21)

Missing n = 9.

#### Foundation Year 2 specialty choice

81/105 respondents had applied for specialty training. 77 respondents reported both their Year 4 and FY2 choices. 34/77 had applied to more than one specialty, 13/34 had a second choice in the same category as their first choice. We refer to respondents’ preferred (first) choice only.

**Figure 1:**
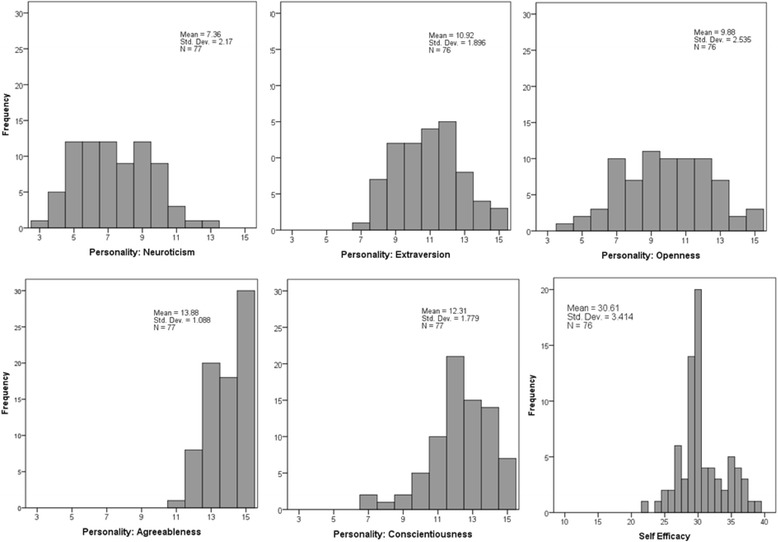
**Distributions and descriptive statistics for personality factors and the self-efficacy variable.**

**Figure 2:**
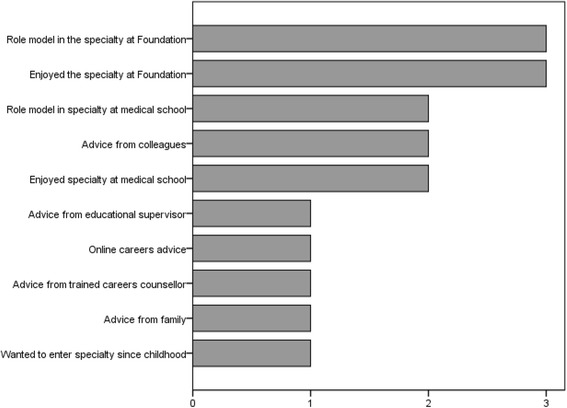
**Median ratings of how influential each experience was in choosing a specialty post-graduation.**

Descriptive statistics for categorical and ordinal questionnaire variables are reported in Table 3. Distributions and descriptive statistics for continuous questionnaire variables are reported in Figure 1. The reported influence that 10 experiences had on participants’ specialty choices is shown in Figure 2.

**Table 3:** **Descriptive statistics (with percentages in parenthesis) for the categorical and ordinal questionnaire variables**

Variable	Descriptive statistics (percentages)					
Stability of choice from year 4 to foundation year 2	50/77 (65) same specialty			27/77 (35) different specialty		
Confident preferred specialty ‘right for me’	22/77 (29) very confident	42/77 (55) confident	10/77 (13) neutral		2/77 (3) unconfident	1/77 (1) very unconfident
Confident will get a job in preferred specialty	3/77 (4) very confident	35/77 (46) confident	31/77 (40) neutral		8/77 (10) unconfident	0/77 (0) very unconfident
Confident will get a job in any specialty (missing = 1)	7/77 (9) very confident	36/77 (47) confident	28/77 (36) neutral		5/77 (7) unconfident	0/77 (0) very unconfident
Considered leaving medicine in the past year (missing = 2)	41/105 (39) never	40/105 (39) once or twice	15/105 (15) monthly		5/105 (5) weekly	2/105 (2) daily
Specialty choice and favourite medical school placement	48/77 (62) same specialty			29/77 (38) different specialty		
Specialty choice and worst medical school placement	2/77 (3) same specialty			75/77 (97) different specialty		
When first considered specialty (missing = 2)	9/77 (12) before medical school	42/77 (54) medical school	17/77 (22) Foundation Year 1		7/77 (9) Foundation Year 2	n/a
When definitely decided on specialty	1/77 (1) before medical school	12/77 (16) medical school	29/77 (38) Foundation Year 1		27/77 (35) Foundation Year 2	8/77 (10) still undecided
Dissuaded from entering a specialty by experiences in the specialty (missing = 2)	13/105 (13) definitely not dissuaded	27/105 (26) probably not dissuaded	8/105 (8) uncertain		32/105(31) probably dissuaded	23/105 (22) definitely dissuaded

### Predictors of stability of choice

#### Specialty chosen

*Stability* varied significantly by specialty (*χ*2 = 17.99; df = 7; p = 0.012). See Table 4.

**Table 4:** **Stability of specialty choice from year 4 medical school to foundation year 2 post-graduation**

FY2 specialty choice	Year 4 specialty choice		Percent (bootstrapped 95% confidence interval)
Paediatrics	Different	0	0
	**Same**	**4**	**100.0 (100.0, 100.0)**
General practice	Different	4	16.0 (4.0, 32.0)
	**Same**	**21**	**84.0 (68.0, 96.0)**
O&G	Different	1	25.0 (0.0, 75.0)
	**Same**	**3**	**75.0 (25.0, 100.0)**
Medical specialties	Different	5	27.8 (11.1, 50.0)
	**Same**	**13**	**72.2 (50.0, 88.9)**
Surgical specialties	Different	4	57.1 (16.7, 87.5)
	**Same**	**3**	**42.9 (12.5, 83.3)**
Psychiatry	Different	4	66.7 (33.3, 100.0)
	**Same**	**2**	**33.3 (0.0, 66.7)**
Acute care	Different	8	66.7 (38.5, 92.3)
	**Same**	**4**	**33.3 (7.7, 61.5)**

Radiology and public health excluded as only one respondent chose each. Bootstrapped 95% confidence intervals for the percentages show Paediatrics was significantly more stable than all specialties except O&G, and General Practice was significantly more stable than Psychiatry and Acute Care.

#### Experiences

Respondents with a stable choice were more influenced by enjoying the specialty at medical school [median ‘stable choice’ = 3 (bootstrapped 95% CI = 2,3); median ‘different choice’ = 2 (bootstrapped 95% CI = 2,3); Mann Whitney U: z = −1.7; p = .08].

Respondents whose choice changed were more influenced by enjoying the specialty since qualifying [median ‘stable choice’ = 3 (bootstrapped 95% CI = 2,3); median ‘different choice’ = 3 (bootstrapped 95% CI = 3,3); Mann Whitney U: z = −2.1; p = 0.03].

#### Being dissuaded from choosing a specialty

Respondents with a stable choice were *less* likely to have been dissuaded [not dissuaded: ‘stable choice’ = 51% (bootstrapped 95% CI = 37, 65); ‘different choice’ = 26% (bootstrapped 95% CI = 10,44); Mann Whitney U: z = −1.8; p = 0.078] [‘stable choice’ median = 2 (‘probably not dissuaded’) (bootstrapped 95% CI = 2,4); ‘different choice’ median = 4 (‘probably dissuaded’) (bootstrapped 95% CI = 3,4)].

#### When first considered specialty

Respondents whose choice changed were more likely to have first considered their specialty in Foundation Year 1 [‘stable choice’ = 12% (bootstrapped 95% CI = 4, 22); ‘different choice’ = 44% (bootstrapped 95% CI = 26,64)]; *χ*^2^ = 10.6; df = 3; p = 0.014].

No other factors were significantly univariately related to *Stability*.

#### Regression

Specialty chosen was the only significant independent predictor of *Stability*. Those who chose psychiatry were more likely than those who chose general practice to change their choice [B = −3.81 (bootstrapped 95% CI = −27.76, −0.95); odds ratio = 0.003; bootstrapped p = 0.02]. See Table 5. Paediatrics split the outcome variable completely (all those who chose paediatrics had a stable choice), so it was excluded from the regression.

**Table 5:** **Factors related to stability of specialty choice from Year 4 medical school to Foundation Year 2 (0 = ‘different choice; 1 = ‘stable choice’)**

Predictors		B (bootstrapped 95% CI)	Bootstrapped standard error	p value	Bootstrapped p value	Odds ratio
(1 = before medical school; 2 = at medical school; 3 = at FY1; 4 = at FY2)	First considered specialty	−0.54 (−2.49, 0.93)	1.60	0.278	0.58	0.310
(1 = no influence; 2 = moderate influence; 3 = strong influence)	Enjoyed specialty at medical school	0.95 (−0.45, 3.71)	2.58	0.089	2.57	0.121
Reference category: General Practice (Paediatrics & Radiology excluded)	Acute care	−2.24 (−20.19, 0.28)	6.05	0.026	0.11	0.027
	Medical specialties	−0.53 (−4.32, 2.36)	3.98	0.553	0.59	0.612
	Surgical specialties	−2.66 (−24.71, 0.52)	8.52	0.023	0.07	0.019
	Obstetrics & Gynaecology	−1.14 (−22.19, 20.07)	12.31	0.423	0.32	0.218
	**Psychiatry**	−3.81 (−27.76, −0.95)*	**11.96**	**0.008**	**0.02**	**0.003**
(1 = not dissuaded; 2 = probably dissuaded; 3 = uncertain; 4 = probably not dissuaded; 5 = not dissuaded)	Dissuaded from entering a specialty by experienced since starting work	−0.33 (−1.31, 0.21)	0.42	0.170	0.72	0.215
(1 = no influence; 2 = moderate influence; 3 = strong influence)	Enjoyed the specialty since starting work	−0.77 (−3.00, −0.03)	1.79	0.057	0.46	0.046
	Constant	3.83 (−1.37, 15.89)	6.69	0.102	46.23	0.115

Bootstrapping based on 976 samples. *significant at p <0.05 in a logistic regression.

### Differences between respondents who chose different specialties

#### Demographics and specialty chosen

General practice was more popular with black and minority ethnic doctors than surgical specialties, O&G, and paediatrics, which were not chosen by any BME respondents [GP: 80% white (bootstrapped 95% CI = 60,96)].

#### Confidence and specialty chosen

Respondents who chose surgical specialties (mean confidence = 2.8; bootstrapped 95% CI = 2.4, 3.4) were less confident that they would get a job in their preferred specialty compared to respondents who chose medical specialties (mean confidence = 3.7; bootstrapped 95% CI = 3.3,4.0) and psychiatry (mean confidence = 3.7; bootstrapped 95% CI = 3.2,4.0) [F(6,74) = 2.5; p = 0.028].

#### When decided to enter specialty

Respondents who chose surgical specialties on average made their decision at medical school, whereas all others except O&G made their decision later or were still undecided [median surgical specialties = 2 (bootstrapped 95% CI = 2,2); median O&G = 3.5 (bootstrapped 95% CI = 1,4); median all other specialties range = 3.5 to 4 (bootstrapped 95% CI range = 3 to 5); *χ*^2^ = 14.5; df = 6; p = 0.024].

#### Perceptions of the specialty

The ‘lifestyle’ factor was significantly more important to those who chose general practice (mean = 1.0; bootstrapped 95% CI = 0.8, 1.3) than those who chose surgical specialties (mean = −0.9; bootstrapped 95% CI = −1.3, −0.5), O&G (mean = −0.7; bootstrapped 95% CI = −1.2, −0.5), and medical specialties (mean = −0.4; bootstrapped 95% CI = −0.7,-0.3), [F(6,64) = 13.5; p < 0.001].

The negative ‘had to do something’ factor was significantly more important to those who chose psychiatry (mean = 1.3; bootstrapped 95% CI = 0.1,2.9) than those who chose surgery (mean = −0.4; bootstrapped 95% CI = −0.6,-0.2), [F(6,64) = 2.3; p = 0.043].

The factor ‘using one’s intellect to help others was significantly more important to those who chose psychiatry (mean = 0.8; bootstrapped 95% CI = 0.5,1.1) than those who chose general practice (mean = −0.5; bootstrapped 95% CI = −0.10,-0.06); [F(6,64 = 2.07; p = 0.053].

#### Personality

Respondents who chose acute care (mean = 12.7; bootstrapped 95% CI = 11.8,13.6) were significantly more extroverted than those who chose medical specialties (mean = 10.8; bootstrapped 95% CI = 10.0,11.5), psychiatry (mean = 10.5; bootstrapped 95% CI = 9.3,11.7), and general practice (mean = 10.0; bootstrapped 95% CI = 9.4,10.6) [F(6,67) = 4.03; p = 0.002].

No other factors differed significantly between respondents choosing difference specialties at FY2.

### Predictors of not having chosen a specialty

The 80 respondents who had chosen a specialty (including the three without a Year 4 choice and thus excluded from the *Stability* analyses) were compared to the 25 who had not chosen a specialty.

#### Cohort

More respondents in the 2008 cohort had not chosen a specialty (*χ*2 = 3.9; df = 1; p = 0.048).

#### Satisfaction

Respondents who had not chosen a specialty were twice as likely to have considered leaving medicine monthly or more in the last year [considered leaving monthly or more: no specialty = 38% (bootstrapped 95% CI = 21, 58); specialty chosen = 17% (bootstrapped 95% CI = 8, 25); Mann Whitney U: z = −1.87; p = 0.06].

#### Personality

Respondents who had not chosen a specialty were significantly higher on neuroticism [t(99) = 2.1; bootstrapped p = 0.047; mean difference = 1.2 (95% CI = 0.1,2.3)]; lower on agreeableness [t(99) = −2.6; bootstrapped p = 0.032; mean difference = −0.7 (95% CI = −1.4,-0.1)]; and lower on conscientiousness [t(99) = −3.4;bootstrapped p = 0.002; mean difference = −1.5 (95% CI = −2.4,-0.6)].

#### Regression

Low agreeableness [B = 0.53 (bootstrapped 95% CI = 0.17, 1.20); odds ratio = 1.69; bootstrapped p = 0.028] and low conscientiousness [B = 0.40 (bootstrapped 95% CI = 0.11, 0.86); odds ratio = 1.49; bootstrapped p = 0.004] were the only significant independent predictors of not having chosen a specialty at FY2. See Table 6.

**Table 6:** **Factors related to ‘not choosing a specialty’ (0 = no specialty chosen; 1 = specialty chosen)**

Predictors		B (bootstrapped 95%CI)	Bootstrapped Standard error	p value	Bootstrapped p value	Odds ratio
(reference: 2007 cohort)	2008 cohort	−0.99 (−2.84, 0.17)	0.95	0.093	0.091	0.37
Personality	Neuroticism	−0.10 (−0.51, 0.183)	0.18	0.435	0.484	0.90
	**Agreeableness**	0.53 (0.17, 1.20)*	**0.30**	**0.021**	**0.028**	**1.69**
	**Conscientiousness**	0.40 (0.11, 0.86)*	**0.20**	**0.005**	**0.004**	**1.49**
(1 = monthly or less; 2 = once or twice; 3 = never)	Satisfaction (frequency considered leaving medicine in the past year)	0.55 (−0.59, 1.68)	0.56	0.169	0.253	1.73
	Constant	−10.31 (−23.77, 0.91)	6.230	0.017	0.037	0.00

Bootstrapping based on 1,000 samples. *significant at p<0.05 in a logistic regression.

### Relationship between confidence, personality, and self-efficacy

There was a large correlation between confidence in getting a job in a chosen specialty and confidence in getting a job generally (r = 0.87; bootstrapped 95% CI = 0.77, 0.93).

Respondents who were less extraverted were more confident they would get a job in their chosen specialty (r = −0.28; bootstrapped 95% CI = −0.49, −0.05) and generally (r = −0.28; bootstrapped 95% CI = −0.48, −0.05).

Respondents with higher self-efficacy were lower on neuroticism (r = −0.31; bootstrapped 95% CI = −0.48, −0.13), higher on extraversion (r = 0.4; bootstrapped 95% CI = 0.2, 0.6), and higher on conscientiousness (r = 0.39; bootstrapped 95% CI = 0.18, 0.59). Self-efficacy was not related to confidence.

## Discussion

This longitudinal study found that when considering groups of specialties, two thirds of BSMS graduates had a Foundation Year 2 specialty choice that had been stable since Year 4 of medical school. Unexpectedly stability was not related to confidence but it was related to the specific specialty group chosen. Specialty choice was reportedly strongly influenced by experiences within the specialty at medical school and after starting work. Half of doctors said they had been dissuaded from entering a particular specialty, and only two chose a specialty they had disliked at medical school. Perceptions of the specialty were influential, lifestyle factors were important to those applying for general practice and psychiatry, and those who chose psychiatry were also most likely to say they ‘had to choose something’. General practice was popular with Black and Minority Ethnic doctors, none of who chose surgical specialties, obstetrics & gynaecology, or paediatrics. About a quarter of doctors had not applied for specialty training, which was predicted by low agreeableness and low conscientiousness.

This is the first UK longitudinal study of medical student and junior doctors’ specialty choices since the MMC reforms were made. It gathered data on doctors’ actual specialty choices and prospective data on their interests at medical school, which are less biased than retrospective recollections. The small numbers of graduates from a single medical school and 50% response rate reduces the reliability and generalisability of the results; however, they reflect findings from other studies, suggesting a broadly representative sample [6,19]. All participants had completed a formative career planning exercise at medical school, which may have made them more certain about their choices than other doctors.

The findings support the literature about the influence of experience and role models on specialty choice [20-23]. Stability of choice was not related to confidence or satisfaction generally with medicine, suggesting that change can be positive, particularly if properly supported. Career advice during medical school could encourage students to assemble a tentative short-list of specialties to explore during foundation training [24] rather than to decide on the specific specialty. However, currently applicants need to show ‘commitment to specialty’ by for example assembling audits and attend conferences in that specialty, which encourages early choices. As an alternative, a focus on ‘depth of career planning’ may be more appropriate so that applicants who have changed their minds during the foundation programme are not penalised.

In this study, senior doctors were an importance source of informal career advice, and thus may benefit from careers guidance training, particularly as, in our experience, only a small minority of junior doctors seek in-depth career counselling and usually this is linked to health or performance issues, or to the desire to leave clinical practice.

A quarter of the respondents in this study had not applied for specialty training. Nationally, relatively high numbers of junior doctors take a break after foundation training, often to work abroad as a doctor, but few leave medicine at this point [25]. The significant association between dissatisfaction with medicine and not having applied for specialty training in our study was underpinned by personality, particularly low agreeableness and low conscientiousness. Previous research has found associations between low conscientiousness, low agreeableness, and stress [26], suggesting medical students and junior doctors who experience stress more keenly than others may need additional support with making robust career decisions. Conscientiousness predicts academic performance [27,28], which we did not measure in this study. It may be that those who did not apply were taking time out to improve their CV and improve their chances in subsequent recruitment rounds.

Although related to negative experiences, being put off a specialty can be useful for individuals looking to choose between one or more specialties. Negative experiences may be more problematic for some specialties such as psychiatry [29-31]. In our study, graduates who chose psychiatry scored significantly higher on the negative factor ‘had to do something’, but also on the factor ‘using one’s intellect to help others’, and on the ‘lifestyle’ factor, supporting previous findings [31]. The Royal College of Psychiatrists recommend medical school rotations showcase this specialty more effectively [30]. Initiatives could also emphasise relevant positive aspects such as its potential intellectual challenges and the fact that it is possible to work at consultant level while achieving an acceptable work-life balance.

## Conclusions

Despite changes that require junior doctors to choose a specialty 18 months after graduating from medical school, one of the most important influences on specialty choice remains experience in that specialty during foundation training, including the influence of role models and senior colleagues. Many graduates in our sample also had a good idea of broad type of specialty they would like to enter while still at medical school, and rated medical school experiences as highly influential. Taken together, these findings highlight the importance of starting career guidance for doctors at medical school, but also continuing it through the foundation years when final decisions are cemented. They suggest greater emphasis should be placed on ensuring that trainees have access to a wide variety of specialties, and that senior clinicians are provided with support in advising junior colleagues, particularly those with a tendency to stress. Future research could investigate the impact of career planning interventions at medical school by following up doctors who had received such interventions with those who had not, and looking at whether stability of choice from medical school to post-foundation training relates to success, both psychological and in terms of performance.
